# Amygdala 14-3-3ζ as a Novel Modulator of Escalating Alcohol Intake in Mice

**DOI:** 10.1371/journal.pone.0037999

**Published:** 2012-05-22

**Authors:** Heidi M. B. Lesscher, Julia M. Houthuijzen, Marian J. Groot Koerkamp, Frank C. P. Holstege, Louk J. M. J. Vanderschuren

**Affiliations:** 1 Rudolf Magnus Institute of Neuroscience, Department of Neuroscience and Pharmacology, University Medical Center Utrecht, Utrecht, The Netherlands; 2 Division of Behavioural Neuroscience, Department of Animals in Science and Society, Faculty of Veterinary Medicine, Utrecht University, Utrecht, The Netherlands; 3 Molecular Cancer Research, University Medical Center Utrecht, Utrecht, The Netherlands; Radboud University, Netherlands

## Abstract

Alcoholism is a devastating brain disorder that affects millions of people worldwide. The development of alcoholism is caused by alcohol-induced maladaptive changes in neural circuits involved in emotions, motivation, and decision-making. Because of its involvement in these processes, the amygdala is thought to be a key neural structure involved in alcohol addiction. However, the molecular mechanisms that govern the development of alcoholism are incompletely understood. We have previously shown that in a limited access choice paradigm, C57BL/6J mice progressively escalate their alcohol intake and display important behavioral characteristic of alcohol addiction, in that they become insensitive to quinine-induced adulteration of alcohol. This study used the limited access choice paradigm to study gene expression changes in the amygdala during the escalation to high alcohol consumption in C57BL/6J mice. Microarray analysis revealed that changes in gene expression occurred predominantly after one week, i.e. during the initial escalation of alcohol intake. One gene that stood out from our analysis was the adapter protein 14-3-3ζ, which was up-regulated during the transition from low to high alcohol intake. Independent qPCR analysis confirmed the up-regulation of amygdala 14-3-3ζ during the escalation of alcohol intake. Subsequently, we found that local knockdown of 14-3-3ζ in the amygdala, using RNA interference, dramatically augmented alcohol intake. In addition, knockdown of amygdala 14-3-3ζ promoted the development of inflexible alcohol drinking, as apparent from insensitivity to quinine adulteration of alcohol. This study identifies amygdala 14-3-3ζ as a novel key modulator that is engaged during escalation of alcohol use.

## Introduction

Alcoholism, characterized by a loss of control over alcohol intake, is a disease that affects over 76 million people worldwide [1]. Although recent years have seen progress in this regard, treatment strategies for alcoholism are still limited in number and efficacy [2]–[4], which underscores the pressing need to understand the neural underpinnings of alcoholism.

The development of alcoholism is caused by alcohol-induced maladaptive changes in neural circuits involved in emotions, motivation, habit formation and decision making [5]–[11]. The amygdala is a key structure in several of these processes. Within the amygdala, integration of sensory information and attribution of affective valence to primary rewards and associated cues takes place. Connections of the amygdala with the nucleus accumbens, ventral tegmental area and prefrontal cortex allow amygdaloid mechanisms to influence various aspects of alcohol-motivated behavior, while projections from the amygdala to hypothalamus and brainstem contribute to arousal and stress that promote alcohol intake [12]–[18].

Human alcoholics have reduced amygdala volumes [19] and exposure of alcoholics to alcohol odor induces intense craving that is associated with amygdala activation [20]. Consistent with its role in processing negative emotional stimuli, the amygdala contributes to alcohol consumption in alcohol-dependent animals that display enhanced negative affect. Increased alcohol intake and enhanced negative affect, apparent from decreased brain reward and enhanced anxiety-like behavior, in alcohol-dependent animals is associated with neurophysiological changes in the amygdala including enhanced CRF and GABA release [21]–[23]. These observations are consistent with the known involvement of the amygdala in the generation and perception of positive and negative emotions [17], [24], as well as in the influence of behaviorally meaningful environmental cues, such as drug-associated conditioned stimuli, on behavior [13], [16], [17], [25]–[27]. Recent evidence has implicated physiological changes in the central nucleus of the amygdala (CeA) in alcohol intake. Thus, escalation of alcohol intake and the development of alcohol dependence has been shown to be paralleled by alterations in neuropeptide expression and changes in GABAergic neurotransmission [28], [29] and to involve PKC signaling [30], but the molecular mechanisms involved remain incompletely understood.

The aim of this study was to identify molecular mechanisms in the amygdala that contribute to the escalation of alcohol intake, which is an important stage in the development of drug addiction [31]. During the development of alcoholism, casual alcohol use escalates into excessive drinking, ultimately culminating in full-blown alcohol addiction, characterized by loss of control over alcohol intake. For this study we used a limited access choice paradigm, in which C57BL/6J mice show rapid escalation of alcohol consumption [32], which depends on processes within the CeA [30]. Moreover, using this paradigm C57BL/6J mice display alcohol use despite adverse consequences, an important behavioral characteristic of alcoholism, in that they fail to reduce their alcohol intake when an alcohol solution is adulterated with quinine and consume an aversive, quinine-containing alcohol solution despite the simultaneous availability of unadulterated alcohol [33]. Here, we studied gene expression patterns in the CeA during the escalation of alcohol intake in C57BL/6J mice, using microarray analysis followed by qPCR. We subsequently used RNA interference to pinpoint the involvement of 14-3-3ζ, a candidate gene that stood out from our analysis, in alcohol intake. Together, these data show that 14-3-3ζ signaling in the CeA controls the escalation of alcohol intake in mice.

## Materials and Methods

### Animals

8–10 Weeks old male C57BL/6J mice, derived from Jackson Labs (Bar Harbor, Maine, USA) and bred in our facility, were group-housed with food and water *ad libitum* under controlled conditions (20±2°C and 50–70% humidity) and acclimatized to a 12-h light/dark cycle (7:00 AM lights off) at least 2 weeks prior to testing. Experimental procedures were approved by the Animal Ethics Committee of Utrecht University and conducted in agreement with Dutch laws (Wet op de dierproeven, 1996) and European regulations (Guideline 86/609/EEC).

### Limited access alcohol consumption

Mice were trained to voluntarily consume alcohol using a limited access choice paradigm [30], [34], [35]: they had access to one drinking tube containing tap water and one containing alcohol (10–15% v/v) in daily 2 hour sessions, starting 3 hours into the dark cycle. Bottle positions were switched daily after 7 days to avoid side-preference. Fluid volumes were measured and alcohol intake, alcohol preference and total volume consumed were calculated.

For microarray analysis, mice were randomly assigned to 3 experimental groups that consumed alcohol for 1, 2 or 4 consecutive weeks (*N* = 14, 14 and 13), representing 3 stages of the development of alcoholism: initial escalation of alcohol intake (1 week), the stage where high alcohol intake is reached and insensitivity to quinine adulteration emerges [33] and the stage of stable high alcohol intake (4 weeks). A water control group (*N* = 6) was included and a group of naïve mice (*N* = 21) served as a reference sample. To ensure active engagement in the limited access choice paradigm and to exclude animals consuming extremely high or low amounts of alcohol, we applied the following inclusion criteria.

1. *To ensure sufficient sampling of the fluids*, total fluid intake should be ≥12 ml/kg on day 6 [30], [32].
2. *To ensure active engagement and motivation to consume alcohol*, preference for alcohol over water should be >50% by day 6–8.
3. *To ensure analysis of escalation,* alcohol intake should be ≤2 g/kg by day 4 and ≥0.6 g/kg by day 6–8.

The final sample size after application of these criteria was *N* = 6.

For qPCR validation, a separate batch of mice consumed alcohol for 1 week or 2 weeks (*N* = 13); a total of 6 and 7 mice, respectively, met the inclusion criteria. Naïve mice (*N* = 7) and a water group (*N* = 6) were included as controls.

### Tissue dissection

The mice were sacrificed by decapitation 10–11 hours after the final limited access choice session. Blood alcohol analyses using an NAD/ADH assay (Sigma, Germany) confirmed clearance of alcohol from blood at this time: blood alcohol levels were low (6.5–35 mg/dl) compared to levels of 97.7±24.9 mg/dl immediately after a drinking session [32]. Brains were dissected, snap frozen on dry ice and stored at −80°C. Amygdala samples were obtained using a 20G punch needle, aiming at the CeA [30], [36] and were immersed instantly in RNAlater (Sigma, Germany). Total RNA was isolated from the amygdala using TRIzol (Invitrogen, NL), DNAse treated (Ambion, TX, USA) and purified using the RNeasy MinElute Cleanup kit (Qiagen N.V., NL). RNA integrity was confirmed using the Bioanalyzer (Agilent Technologies Inc, CA, USA).

### RNA isolation and Hybridization

Two-color oligonucleotide microarray analysis was performed as described [37]. RNA was amplified in a single round and complementary DNA (cDNA) was synthesized with Superscript III reverse transcriptase (Invitrogen) using a T7 oligo(dT)24VN primer [38]. Complementary DNA was transcribed *in vitro* using the T7Megascript kit (Ambion) in the presence of aminoallyl-UTP, and copy ribonucleic acid (cRNA) quality was evaluated using the Bioanalyzer. Cy3 or Cy5 fluorophores (Amersham Biosciences, NL) were coupled to 1500 ng cRNA, and label incorporation was monitored by spectrophotometry and hybridizations were set up with 1000 ng of Cy3-labeled and 1000 ng of Cy5-labeled cRNA. Each cRNA sample was labeled with Cy3 or Cy5 and was hybridized in dye swap against a common reference pool sample consisting of RNA from naïve mice; a total of 6 slides were hybridized for each experimental group. The mouse Array-Ready oligo set (version 3.0; Operon Biotechnologies GmbH, Germany) was printed on Corning UltraGAPS slides as previously described [37]. Slides were washed manually, scanned in the Agilent G2565AA DNA Microarray Scanner (100% laser power and 30% photomultiplier tube) and quantified and background corrected with IMAGENE (version 5.6.1; BioDiscovery, Inc., CA, USA) and Loess normalized per print-tip [39].

The microarray data were analyzed by ANOVA-modeling [40] to identify genes that show differential expression from naïve control mice. In a fixed effect analysis, sample, array and dye effects were modeled. Sample-specific differences between groups were then modeled and tested using permutations and family-wise error correction. MIAMEcompliant descriptions of protocols, experiment design, arrays, raw and normalized data have been deposited in the public microarray database ArrayExpress (http://www.ebi.ac.uk/arrayexpress/), all under the experiment accession number E-TABM-956. In addition, the microarray data was analyzed using the short time-series expression miner (STEM) with integrated gene ontology (GO) database [41].

### qPCR Validation

Total RNA was isolated from the amygdala as described and cDNA was synthesized from the RNA samples using oligo-dT primers. qPCR analysis was performed using the LightCycler (Roche, NL), the Fast Start DNA Master PLUS SYBRgreen I kit (Roche) and primers listed in Table 1. After initial normalization to the housekeeping gene beta-actin, gene expression was calculated as the ratio to levels of naïve mice using the comparative Ct method [42].

**Table 1:** Primer sequences for qPCR validation.

Gene	Primer Fwd	Primer Rev
Gabrg2	TGTGGTCACCGAATGTGTTT	TCATTGCTGTTGCTCAAAGG
Gria3	CTCGGTGCTTTCCTAAAACG	CCAAACACGTCTGGGAGAAT
YWHAZ	AGCAGGCAGAGCGATATGAT	TTCTCAGCACCTTCCGTCTT
Gprasp1	CCCATTAGATCCCCTTGGTT	CCTGTGATGGTCTTGGTCCT
Ctnnd2	CGCCAGCATCACTTGTCC	ACTGCTTCCTGGCGAACAT
Tmod2	CCAGCTAAATTGTGGGCATT	GGCTCTGTCTTCGAGGTGAC
Gabrb3	AGGCATCCATAAACCGACTG	GGCCACCGAAAAATCAAGTA
Prkacb	TCTTTCCTGCGTCATCAGTG	AAGGGAGCACTGGTCAGAGA
Actb (beta-actin)	AGCCATGTACGTAGCCATCC	CTCTCAGCTGTGGTGGTGAA

### RNA interference

Two shRNAs were designed for mouse 14-3-3ζ (YWHAZ, NCBI accession no. NM_011740): 1222 bp (GTGAAGAGTCGTACAAAGG) and 1854 bp (GAAGTTGTCTCTAGACAAG). A non-coding sequence was used as a control (GCGTGTACGGACCTATTGG). The sequences were cloned into a LentiLox 3.7 vector (pLL3.7, http://www.sciencegateway.org/protocols/lentivirus/pllmap.html).

Knockdown efficiencies were determined by transfection of Neuro2A cells with the respective lentiviral vectors and semi-quantitative 14-3-3ζ protein analysis by western blot 48 hours after transfection. For each condition 5, 10, 15 and 20 μg protein was loaded and 14-3-3ζ was visualized using goat anti-14-3-3ζ antibody (SantaCruz, 1∶5000), donkey anti-goat HRP (Jackson ImmunoResearch, 1∶25.000) and detection by enhanced chemiluminescence (Thermo Scientific). Knockdown efficiency was determined by densitometry and comparative slope analysis as described [30].

Lentivirus was produced by co-transfecting HEK293T cells with the respective shRNA-pLL3.7 vector (22.5 μg), pMD2\VSV-G (7.9 μg), pMDL\pRRE (15.6 μg) and pRSV-Rev (5.6 μg). Viral titers were determined in Neuro2A cells and expressed as number of infected GFP-positive cells per viral volume.

### *In vivo* knockdown of 14-3-3ζ in the CeA and alcohol intake

To establish *in vivo* knockdown of 14-3-3ζ, male C57BL/6J mice were anaesthetized with ketamine (75 mg/kg i.p.) and medetomidine (1 mg/kg i.p.) and placed in a stereotaxic frame (David Kopf Instruments, CA, USA). The injectors (33G) were targeted at the CeA using the coordinates: −0.90 mm posterior to bregma, +/− 3.0 mm lateral to midline and −4.6 mm ventral from bregma [36]. Lentivirus (2 μl, 3×10^7^ iU/ml) was infused at a rate of 0.2 μl/min.

To determine knockdown efficiency *in vivo*, control lentivirus was infused in one hemisphere and lentivirus expressing 1854 14-3-3ζ shRNA was infused into the contralateral CeA. Three weeks after infection, *in situ* hybridization for 14-3-3ζ and GFP was performed using digoxigenin-labeled cRNA probes transcribed from mouse 14-3-3ζ (1046 bp fragment) and eGFP (720 bp fragment) cDNAs as described [43] and mRNA levels were compared within animals (*N* = 3).

For alcohol consumption experiments, control or 14-3-3ζ shRNA expressing lentivirus was infused bilaterally into the CeA. Alcohol consumption was determined in the limited access choice paradigm as described (*N* = 8–9) after 3 weeks post-surgery recovery and adaptation to the reversed light-dark cycle (7:00 AM lights off). Inflexible drinking behavior was assessed by adulterating alcohol (15% v/v) with graded quinine concentrations (100 μM, 250 μM, 350 μM, 500 μM and 750 μM) on 5 consecutive days [33]. Alcohol intake and preference were normalized to the group average over the last 3 days prior to quinine modulation. After completion of the alcohol consumption, intake of sweet (sucrose / saccharin) and bitter (quinine) solutions was also determined in two-bottle choice tests.

Post-mortem immunohistochemistry for GFP was performed using an anti-sheep antibody (1∶5000, Biogenesis Ltd) to determine the infection site.

### Data Analysis

*Consumption data* were analyzed by one-way repeated measures ANOVA with group as the between-subjects factor and time or quinine concentration as the repeated measures within-subjects factor. *qPCR data* were analyzed by one-way ANOVAs with group as the between-subjects factor, followed by Tukey HSD multiple comparisons. Post-hoc analysis was performed by two-tailed *t*-tests where appropriate. Differences between pairs of means were considered significant at alpha < 0.05. SPSS 15.0 was used for data analysis.

## Results

### Gene expression during the escalation of alcohol intake

**Figure 1:**
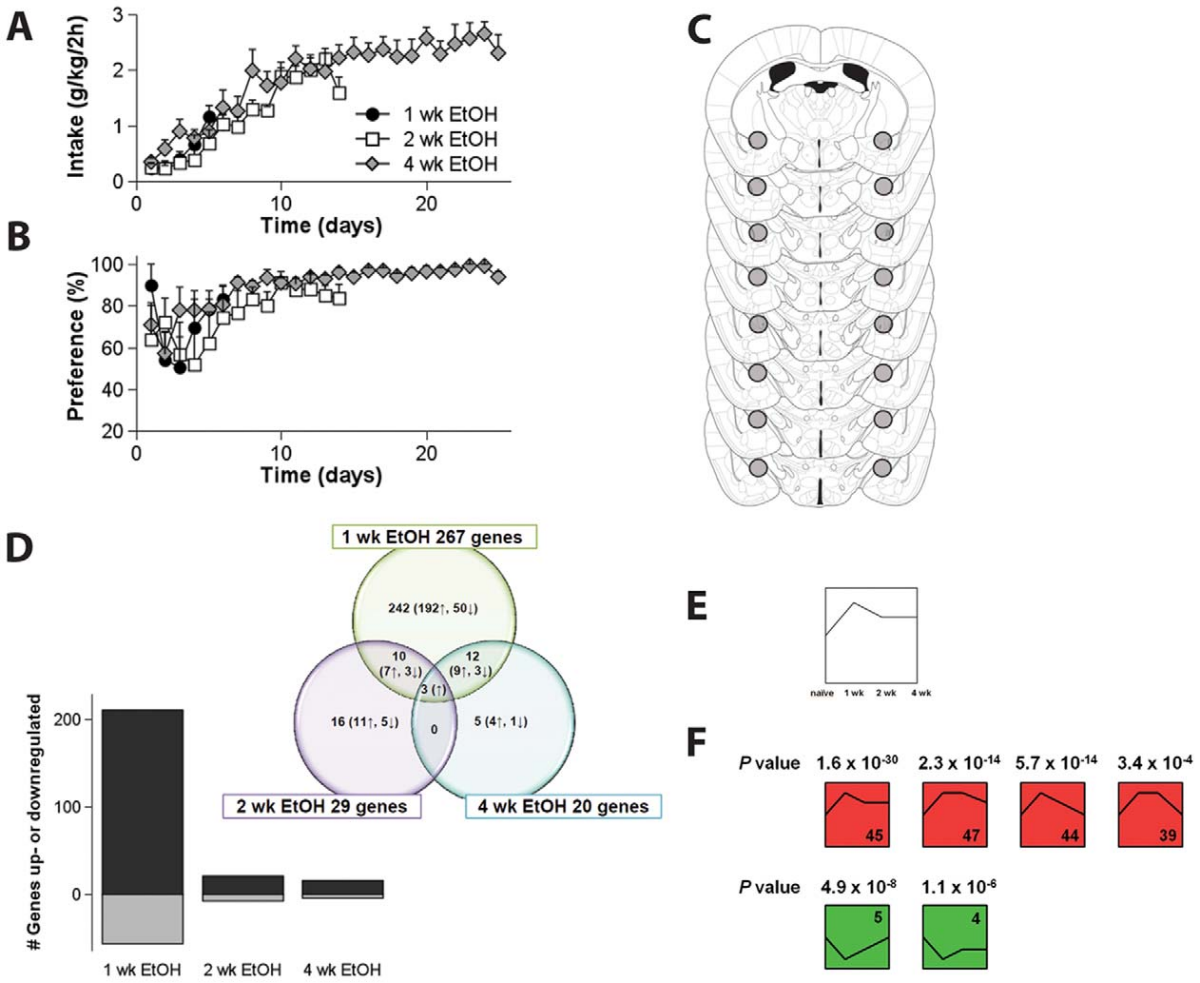
Alcohol intake and preference for C57BL/6J mice that consumed alcohol for 1, 2 or 4 weeks. ***A*** The mice show a progressive increase in alcohol intake during the first two weeks of the experiment, stabilizing thereafter. ***B*** Preference for alcohol was high throughout the experiment. ***C*** RNA for microarray analysis was isolated from 0.6 mm diameter punches (**○**) from serial sections through the CeA. ***D*** The majority of the gene expression changes occurred after 1 week of alcohol consumption; the bar graph shows the total numbers of up- (black) and down-regulated (grey) genes for each time-point while the Venn Diagram shows the total number of up- and down-regulated genes (marked by arrows) for each time-point and overlap between the experimental groups. ***E–F*** Analysis of the microarray data using the *Short Time Series Expression Miner (STEM*), revealed significant gene enrichment particularly during the early stages of escalation of alcohol intake (week 1 and/or week 2). When considering all possible time profiles for gene expression after 1 wk, 2 wk and 4 wk of alcohol consumption, relative to data from naïve control mice (exemplified by ***E***), 6 time profiles showed significant gene enrichment: they represented more genes than expected based on chance (***F***). The commonality between these 6 significant time profiles is that they all show an initial change in gene expression, normalizing thereafter to baseline levels.

In order to trace molecular mechanisms engaged during the escalation of alcohol intake, gene expression in the CeA was studied by microarray analysis after 1 week, 2 weeks and 4 weeks of daily alcohol consumption (Fig. 1*A–B*). Alcohol preference was high from the first week of the experiment onwards (1 week F_(time)5,65_ = 1.7, N.S.; 2 weeks F_(time)7,70_ = 0.97, N.S.; 4 weeks F_(time)10,50_  = 1.1, N.S.) while alcohol intake stabilized only after two weeks of daily drinking. The experimental groups represent 3 stages of escalation to alcoholism-like behavior: the transition from low to high alcohol intake (1 week; intake: F_(time)5,75_ = 16.8, P<0.001), the stage when mice reach their highest levels of alcohol intake and start to show inflexible and indifferent alcohol intake (2 weeks; see Lesscher *et al*, 2010; intake: F_(time)7,70_ = 10.3, P<0.001) and finally also the stage where high alcohol intake has stabilized (4 weeks; intake: F_(time)10,50_ = 0.87, N.S.). Microarray analysis revealed marked changes in gene expression as a result of alcohol consumption, particularly during the early stages of alcohol intake (P<0.01 from control mice, Fig. 1*D* and Fig. 1*E–F*). After exclusion of those genes that showed differential expression after water consumption compared to naïve mice, 267 genes in the CeA were found to be differentially regulated after 1 week of alcohol consumption, i.e. during the transition from low to high alcohol intake. Of these 267 genes, 211 were up-regulated and 56 were down-regulated. By contrast, only 29 and 20 genes, respectively, were differentially regulated after 2 and 4 weeks of alcohol consumption, i.e. when highest alcohol levels are reached and signs of alcoholism-like behavior emerge, and alcohol intake is stabilized. Regulated genes in all three experimental groups are presented in Table 2.

**Table 2:** Significant effects of 1 week, 2 weeks and 4 weeks of daily alcohol consumption in the limited access choice paradigm on gene expression changes in the amygdala of C57BL/6J mice.

Symbol	Gene description	p value	EtOH/N
***1 week alcohol consumption*** *			
Prkacb	cAMP-dependent protein kinase, beta-catalytic subunit	0,0013	1,44
Ywhaz	14-3-3 protein zeta/delta	0,0001	1,43
Mapk10	Mitogen-activated protein kinase 10	0,0007	1,38
Gprasp1	G-protein coupled receptor-associated sorting protein 1	0,0034	1,36
Rasgrp1	RAS guanyl releasing protein 1	0,0037	1,35
Rdm1	RAD52 motif-containing protein 1	0,0024	1,34
Camk2a	Calcium/calmodulin-dependent protein kinase type II alpha chain	0,0002	1,32
Eif4a2	Eukaryotic initiation factor 4A-II	0,0003	1,31
Trim37	Tripartite motif-containing protein 37	0,0098	1,30
Pdia3	Protein disulfide-isomerase A3 precursor	0,0005	1,30
Slc22a17	solute carrier family 22 (organic cation transporter), member 17	0,0055	1,30
Atp6ap1	Vacuolar ATP synthase subunit S1 precursor	0,0002	1,29
Zfp758	zinc finger protein 758	0,0001	1,28
Arf1	ADP-ribosylation factor 1	0,0034	1,28
Rtn4	Reticulon-4 (Neurite outgrowth inhibitor)	0,0003	1,28
Lgi1	Leucine-rich glioma-inactivated protein 1 precursor	0,0094	1,27
Trim23	GTP-binding protein ARD-1	0,0018	1,26
Cdk5rap2	CDK5 regulatory subunit-associated protein 2	0,0000	1,26
Fbxl3	F-box/LRR-repeat protein 3	0,0079	1,26
Cd47	Leukocyte surface antigen CD47 precursor	0,0038	1,26
Pcmtd1	Protein-L-isoaspartate O-methyltransferase domain-cont protein1	0,0036	1,26
Arhgef9	Rho guanine nucleotide exchange factor 9	0,0016	1,26
Actg2	Actin, gamma-enteric smooth muscle	0,0000	1,26
Ptk2b	Protein tyrosine kinase 2 beta	0,0022	1,25
Homer1	Homer protein homolog 1	0,0003	1,25
Lrp11	Low-density lipoprotein receptor-related protein 11 precursor	0,0008	1,25
Dnajc5	DnaJ homolog subfamily C member 5	0,0016	1,25
Ybx1	Nuclease sensitive element-binding protein 1	0,0024	1,24
Gls	glutaminase isoform 1	0,0079	1,24
Arf1	ADP-ribosylation factor 1	0,0038	1,24
Eif4h	Eukaryotic translation initiation factor 4H	0,0045	1,23
Azin1	Antizyme inhibitor 1 (AZI)	0,0016	1,23
Syn2	Synapsin-2	0,0002	1,23
Gapdh	Glyceraldehyde-3-phosphate dehydrogenase	0,0037	1,23
Lrrc58	Leucine-rich repeat-containing protein 58	0,0011	1,23
Tmod2	Tropomodulin-2	0,0023	1,23
Slc17a7	solute carrier family 17	0,0001	1,23
Wsb2	WD repeat and SOCS box-containing protein 2	0,0033	1,22
Gria3	Glutamate receptor 3 precursor	0,0001	1,22
Hnrpk	Heterogeneous nuclear ribonucleoprotein K	0,0007	1,22
Ube2d3	Ubiquitin-conjugating enzyme E2 D3	0,0001	1,22
Cabp5	Calcium-binding protein 5	0,0000	1,22
Sec23a	Protein transport protein	0,0001	1,22
Ddx3x	ATP-dependent RNA helicase	0,0020	1,22
Gapdh	Glyceraldehyde-3-phosphate dehydrogenase	0,0001	1,22
Fbxl16	F-box/LRR-repeat protein 16	0,0010	1,22
Usp31	MKIAA1203 protein	0,0026	1,22
Pja2	E3 ubiquitin-protein ligase Praja2	0,0000	1,22
Srp54a	Signal recognition particle 54 kDa protein	0,0010	1,22
Igf1r	Insulin-like growth factor 1 receptor precursor	0,0000	1,21
Ube2i	SUMO-conjugating enzyme UBC9	0,0026	1,21
Arl8b	ADP-ribosylation factor-like protein 8B	0,0001	1,21
Gabrb3	Gamma-aminobutyric acid receptor subunit beta-3 precursor	0,0002	1,21
Lpgat1	Acyl-CoA:lysophosphatidylglycerol acyltransferase 1	0,0002	1,21
Mrfap1	MORF4 family-associated protein 1	0,0073	1,21
Gabrg2	Gamma-aminobutyric acid receptor subunit gamma-2 precursor	0,0000	1,21
Txndc13	Thioredoxin domain-containing protein 13 precursor	0,0003	1,21
Rab18	Ras-related protein Rab-18	0,0024	1,21
Kcnma1	Calcium-activated potassium channel subunit alpha-1	0,0001	1,21
Spag9	C-jun-amino-terminal kinase-interacting protein 4	0,0010	1,21
Peg3	Paternally-expressed gene 3 protein (ASF-1)	0,0002	1,21
Pten	Phosphatidylinositol-3,4,5-trisphosphate 3-phosphatase	0,0019	1,21
Synj1	Synaptojanin-1	0,0055	1,21
Slc4a10	Sodium-driven chloride bicarbonate exchanger	0,0016	1,21
Kpna3	Importin subunit alpha-3	0,0015	1,20
Abr	active BCR-related isoform 2	0,0052	1,20
Ccdc132	Coiled-coil domain-containing protein 132	0,0099	1,20
Ap2a2	AP-2 complex subunit alpha-2	0,0002	1,20
Ppp2ca	Serine/threonine-protein phosphatase 2A catalytic subunit alpha	0,0014	1,20
Cap2	Adenylyl cyclase-associated protein 2	0,0002	1,20
Psap	Sulfated glycoprotein 1 precursor (SGP-1)	0,0021	1,20
Elmo2	Engulfment and cell motility protein 2	0,0080	1,20
Kcnip2	Kv channel-interacting protein 2	0,0043	1,20
Atp5k	ATP synthase subunit e, mitochondrial	0,0001	0,81
Mt2	Metallothionein-2	0,0034	0,81
Tsx	Testis-specific protein	0,0016	0,80
Olfr779	Olfactory receptor Olfr779	0,0002	0,79
Olfr806	olfactory receptor 806	0,0085	0,77
***2 weeks alcohol consumption***			
Vsnl1	Visinin-like protein 1	0,0001	1,33
Tmem130	Transmembrane protein 130 precursor	0,0005	1,27
Pdia3	Protein disulfide-isomerase A3 precursor	0,0016	1,26
Ipo7	Importin-7	0,0001	1,24
Camk2d	Calcium/calmodulin-dependent protein kinase type II delta chain	0,0005	1,18
Ranbp6	Ran-binding protein 6	0,0016	1,18
Atp6ap1	Vacuolar ATP synthase subunit S1 precursor	0,0081	1,18
Ppap2b	Lipid phosphate phosphohydrolase 3	0,0083	1,17
Ptprz1	protein tyrosine phosphatase, receptor type Z, polypeptide 1	0,0004	1,17
Zbtb2	zinc finger and BTB domain containing 2	0,0005	1,16
Prkar2b	cAMP-dependent protein kinase type II-beta regulatory subunit	0,0051	1,16
Zdhhc2	Palmitoyltransferase ZDHHC2	0,0072	1,16
Rab6	Ras-related protein Rab-6A	0,0099	1,16
Slc6a1	Sodium- and chloride-dependent GABA transporter 1	0,0016	1,16
Arhgap5	Rho GTPase activating protein 5	0,0029	1,16
Gls	glutaminase isoform 1	0,0028	1,15
Gabrg1	Gamma-aminobutyric acid receptor subunit gamma-1 precursor	0,0049	1,15
Dpp6	Dipeptidyl aminopeptidase-like protein 6	0,0022	1,15
Calcr	Calcitonin receptor precursor	0,0069	1,15
Olfr779	Olfactory receptor	0,0010	0,85
Ap4e1	AP-4 complex subunit epsilon-1	0,0003	0,83
Tesc	Tescalcin	0,0070	0,83
Rpl27a	ribosomal protein L27a	0,0007	0,83
Itpka	Inositol-trisphosphate 3-kinase A	0,0099	0,82
Cck	Cholecystokinins precursor	0,0072	0,81
***4 weeks alcohol consumption***			
Pum2	Pumilio homolog 2	0,0061	1,24
Igf1r	Insulin-like growth factor 1 receptor precursor	0,0030	1,21
Pisd	Phosphatidylserine decarboxylase proenzyme	0,0021	1,20
Pkia	cAMP-dependent protein kinase inhibitor alpha	0,0033	1,19
Ptprz1	protein tyrosine phosphatase, receptor type Z, polypeptide 1	0,0021	1,19
Prepl	Prolyl endopeptidase-like	0,0039	1,18
Peg3	Paternally-expressed gene 3 protein (ASF-1)	0,0058	1,17
Kpna1	Importin subunit alpha-1	0,0003	1,17
Prkacb	cAMP-dependent protein kinase, beta-catalytic subunit	0,0015	1,17
Atp6ap1	Vacuolar ATP synthase subunit S1 precursor	0,0030	1,17
Homer1	Homer protein homolog 1	0,0032	1,16
Ncdn	neurochondrin	0,0032	1,16
Dnm1	Dynamin-1	0,0085	1,16
Rasgrp1	RAS guanyl releasing protein 1	0,0003	1,16
Tmem40	Transmembrane protein 40	0,0059	0.84

* for the 1 week group only those genes are shown that display a change in expression of at least +/−20% from naïve controls. EtOH/N  =  fold change of the EtOH group (1 wk, 2 wk or 4 wk) to naïve controls. Hypothetical genes were excluded from this list.

**Figure 2:**
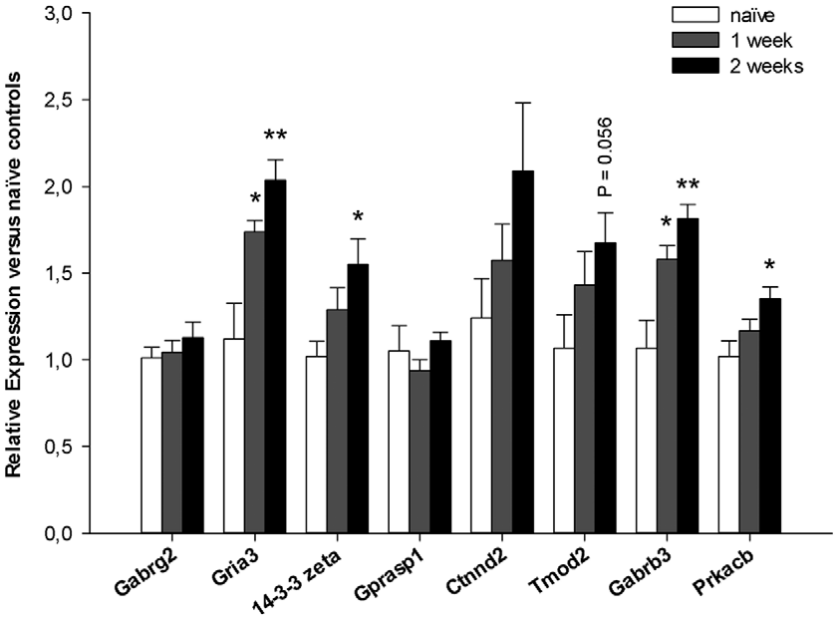
qPCR data for mice that consumed alcohol for 1 week or 2 weeks. qPCR confirmed significant up-regulation of 4 out of 8 candidate genes when compared to naïve control mice: Gria3, 14-3-3 zeta, Gabrb3 and Prkacb. *P < 0.05, **P < 0.01 from naïve mice by Tukey HSD multiple comparisons.

Gene Ontology for those genes that were regulated specifically during the first week of alcohol consumption identified significant enrichment of genes involved in transport, ligand-gated ion channel activity, synaptic transmission and cytoplasm (P_enrichment_ <0.01, Table 3). Based on effect size, significance and evidence for involvement in processes that likely contribute to alcoholism, e.g. synaptic plasticity, memory processing or addiction-related signaling pathways, eight top candidate genes were selected for further investigation. The top candidate genes that we identified include genes which have previously been associated with alcoholism, such as Gabrg2, Gabrb3 and Gria3 [44], [45], but also genes that have not been associated with alcoholism before. Those novel genes are the adapter protein 14-3-3ζ, the G-protein associated sorting protein Gprasp1, two genes involved in structural and functional plasticity (Tmod2 and Ctnnd2) and Prkacb, which has been postulated to affect cAMP-dependent gene expression. qPCR analysis for the 8 top candidate genes in an independent batch of mice confirmed significant up-regulation of Gria3, Gabrb3, 14-3-3ζ and Prkacb in the CeA after alcohol consumption (Fig. 2). Water consumption did not affect the expression of any of the genes tested (*not shown*).

**Table 3:** Gene Enrichment analysis in STEM.

Profile 45				Profile 47	
Transport	Ion transport	Ligand-gated ion channels	Synaptic transmission	Cytoplasm	Protein transport
Acbd5				Apc	
Atp2a2	Atp2a2			Atp2c1	
Atp6ap1	Atp6ap1			B3galnt1	
Camk2a	Camk2a		Camk2a	Caprin1	
			Cspg5	Eps15	Eps15
			**Ctnnd2**	Gatm	
Exoc2				**Gprasp1**	
Exoc5				Ipo7	Ipo7
Gabra1	Gabra1	Gabra1		Itch	
**Gabrb3**	**Gabrb3**	**Gabrb3**		Kif5A	
**Gabrg2**	**Gabrg2**	**Gabrg2**	**Gabrg2**	Mgea5	
Gopc				Mrfap1	
Gria1	Gria1	Gria1		Pdia3	
**Gria3**	**Gria3**	**Gria3**		Peg3	
Kcnip2	Kcnip2			Pppca	
Kif3a				**Prkacb**	
Kpna3					Rab18
Lrp11				Sel1L	
Rab10				Sfrs1	
Slc38a1	Slc38a1			Stx7	Stx7
Snx27				Tm9sf2	
Synj1				Tmed2	Tmed2
			**Tmod2**	Ugcg	
Trappc3				Ybx1	
Trim9			Trim9	14-3-3 η	14-3-3 η
Tsg101				14-3-3 θ	14-3-3 θ
14-3-3ζ					

Gene Ontology analysis for the significant time profiles 45 and 47 in STEM revealed significant enrichment of genes involved in transport, ion transport, ligand-gated ion channel activity, synaptic transmission, cytoplasm and protein transport. Highlighted are the selected **8 top candidate genes**.

### Amygdala 14-3-3ζ controls the development of alcoholism

**Figure 3:**
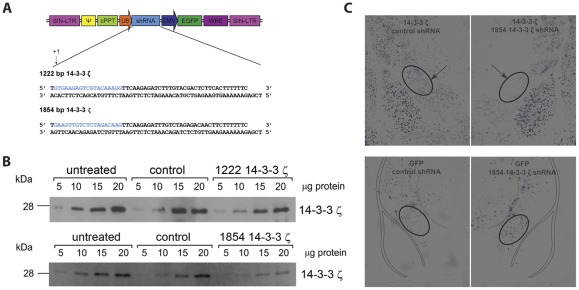
Design and validation of 14-3-3ζ shRNA constructs ***A*** 14-3-3ζ specific shRNA sequences (1222 and 1854 bp) were cloned into pLentiLox3.7 vectors and ***B*** western blot analysis revealed that both constructs effectively reduced 14-3-3ζ protein levels in Neuro2A cells, the 1854 construct being most effective *in vitro.* ***C*** *In situ* hybridization confirmed effective knockdown of 14-3-3ζ in the CeA after infection with the 1854 shRNA expressing lentivirus. The top panels show 14-3-3ζ expression in the CeA after infection with the control or the 1854 shRNA expressing lentivirus. The bottom panels show GFP mRNA and therefore the infection site in adjacent sections. 14-3-3ζ mRNA is completely absent in the area that is infected with the 1854 shRNA expressing lentivirus.

Of the genes that were consistently up-regulated during escalation of alcohol intake, we found 14-3-3ζ to be of particular interest. 14-3-3 Proteins are adapter proteins that have multiple and diverse binding partners [46], [47]. These include transcription factors and signaling molecules such as PKCs [48] and ionotropic glutamate receptors [49] that have previously been implicated in alcoholism [30], [44], [50]-[53]. Therefore, 14-3-3 proteins are well positioned to integrate signaling inputs and influence alcohol addiction. Indeed, previous studies reported gene and protein expression changes for 14-3-3ζ in the nucleus accumbens and amygdala after prolonged alcohol use [54], [55] and a recent study has shown reduced 14-3-3ζ in brain tissue of human alcoholics [56]. Because we found that amygdala 14-3-3ζ levels were up-regulated during the escalation of alcohol intake, we investigated whether amygdala 14-3-3ζ contributes to the escalation of alcohol intake in mice. For this purpose, lentiviral vectors expressing 14-3-3ζ specific shRNA sequences (1222 and 1854 bp, Fig. 3*A*) were generated to reduce the expression of 14-3-3ζ *in vivo*. As a control, a shRNA sequence that did not recognize any known mammalian gene in a BLAST search was used. Of the two shRNA constructs tested, the 1854 construct was most effective in knocking down 14-3-3ζ *in vitro* (Fig. 3*B*). Densitometry and slope analysis revealed lower 14-3-3ζ protein levels in Neuro2A cells that were transfected with both 14-3-3ζ shRNA constructs. Knockdown efficiency, calculated from the ratio of the slopes for 14-3-3ζ shRNA over those for untreated cells, was greater for the 1854 construct (71%) as compared to the 1222 shRNA construct (38%). In contrast, the scrambled construct did not affect 14-3-3ζ protein expression (2.5–3.7% change from untreated cells). *In situ* hybridization after unilateral infusion of the 1854 14-3-3ζ shRNA expressing lentivirus in the CeA and infection with the scrambled shRNA expressing lentivirus in the contralateral CeA confirmed effective knockdown of 14-3-3ζ *in vivo* in the CeA using this same 14-3-3ζ shRNA construct (1854 bp, Fig. 3*C*). Comparison of adjacent sections stained for GFP, to localize the infection site, with sections stained for 14-3-3ζ revealed that 14-3-3ζ mRNA was completely absent in the CeA that was infected with the 1854 14-3-3ζ shRNA expressing lentivirus while 14-3-3ζ was expressed in the contralateral CeA that was infected with the scrambled shRNA expressing lentivirus.

**Figure 4:**
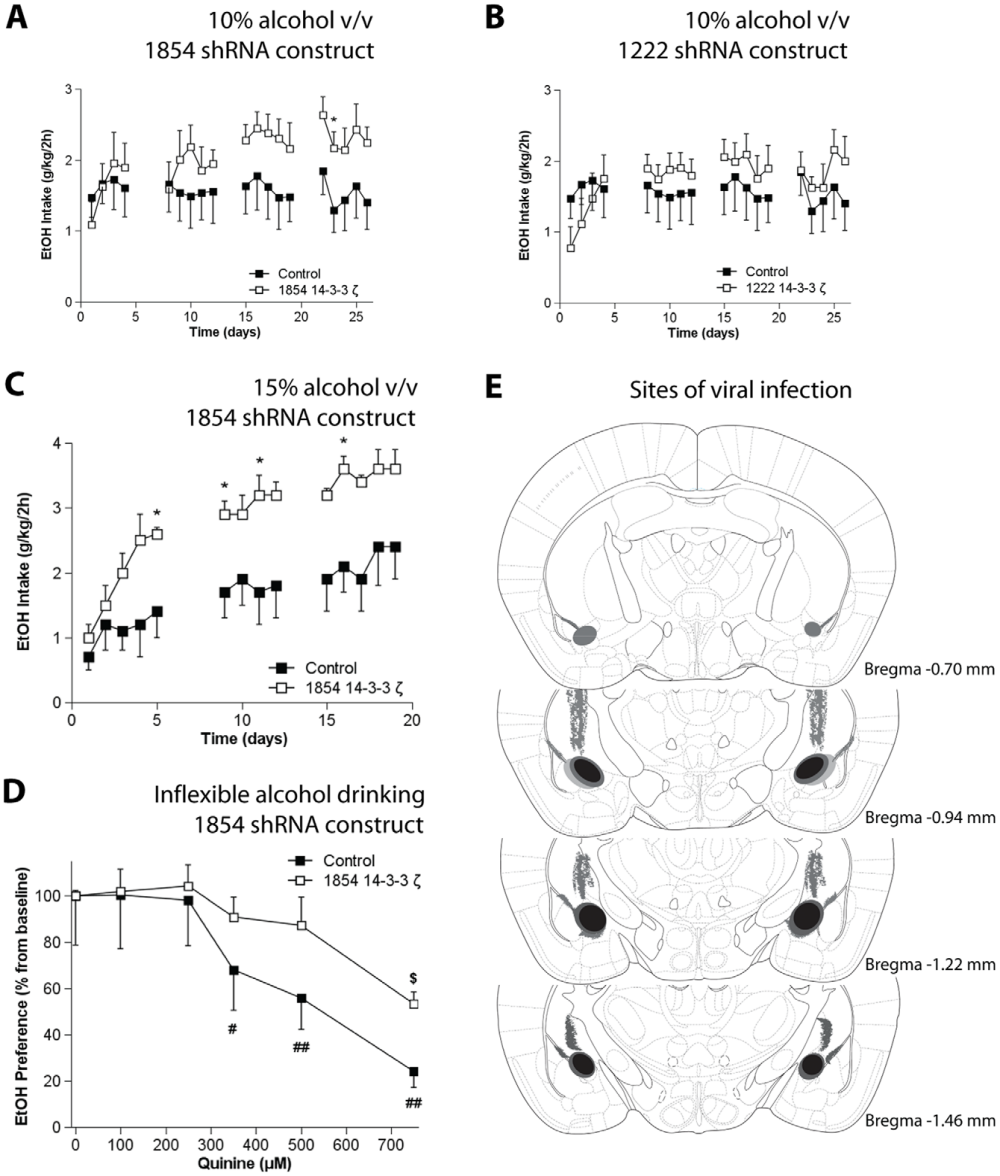
Effects of local knockdown of 14-3-3ζ in the CeA on alcohol consumption. *A* Local knockdown of 14-3-3ζ in the CeA using the 1854 shRNA increased intake of a 10% alcohol solution (v/v). ***B*** Infection with the less effective 1222 shRNA also increased alcohol intake of a 10% alcohol solution (v/v), but less prominently so than the 1854 14-3-3ζ shRNA. ***C*** In a separate batch of mice, local knockdown of 14-3-3ζ in the CeA using the 1854 shRNA increased alcohol intake of a 15% alcohol solution (v/v) and ***D*** local knockdown of 14-3-3ζ in the CeA using the 1854 shRNA caused persistent preference for the alcohol solution despite adulteration with the bitter tastant quinine. In ***E*** the sites of viral infection in the brain are summarized. The black ellipses show the core of the infection site that was consistently targeted across all animals. The areas marked in grey represent less frequent infected sites that include the anterior amygdala, the basolateral amygdala and part of the caudate putamen, along the injection tract. • Control mice; **○** 14-3-3ζ-specific shRNA treated mice. * P<0.05 from controls; # P<0.05, ## P<0.01 from 0 μM quinine for mice treated with control lentivirus; $ P <0.05 from 0 μM quinine for mice treated with the 1854 14-3-3ζ shRNA expressing lentivirus by *t*-test.

The effects of 14-3-3ζ knockdown on escalation of alcohol intake were determined by bilaterally infusing either the 1854 14-3-3ζ or the control lentivirus into the CeA and allowing the mice to consume alcohol in the limited access choice paradigm. The mice with CeA knockdown of 14-3-3ζ using the 1854 14-3-3ζ shRNA construct showed increased alcohol intake (45-53% increase from control in weeks 3 and 4; Fig. 4*A*, 10% v/v; rep. measures ANOVA: F_group(1,12)_ = 1.3, N.S., F_time x group(18,216)_ = 2.0, P<0.05). Mice that were infected with the less effective 1222 14-3-3ζ shRNA expressing lentivirus showed a less prominent but significant increase in alcohol intake (23–22% increase from control in weeks 3 and 4; Fig. 4*B*, 10% v/v; rep. measures ANOVA: F_time x group(18,216)_ = 2.6, P<0.01), demonstrating a gene-dosage effect of amygdala 14-3-3ζ on alcohol intake. We next confirmed the involvement of amygdala 14-3-3ζ in alcohol consumption in a separate batch of mice using a higher alcohol concentration (15% v/v). In agreement with our initial experiment, CeA 14-3-3ζ knockdown using the 1854 shRNA increased intake of the 15% alcohol solution (63–73% increase from control in weeks 1–3; Fig. 4*C*; rep. measures ANOVA: F_group(1,11)_ = 5.1, P<0.05, F_time x group(13,143)_ = 1.0, N.S.). Furthermore, to evaluate the relevance of our findings for alcoholism-like behavior, we next determined the effects of amygdala 14-3-3ζ knockdown on the development of inflexible alcohol drinking [33]. For this purpose, the alcohol solution was adulterated with graded concentrations of the bitter substance quinine. The mice with CeA 14-3-3ζ knockdown using the 1854 shRNA showed a persistent high preference for the quinine-adulterated alcohol solution, indicative of inflexible alcohol drinking (Fig. 4*D*; F_quinine x group(5,50)_ = 2.37, P = 0.053).

**Figure 5:**
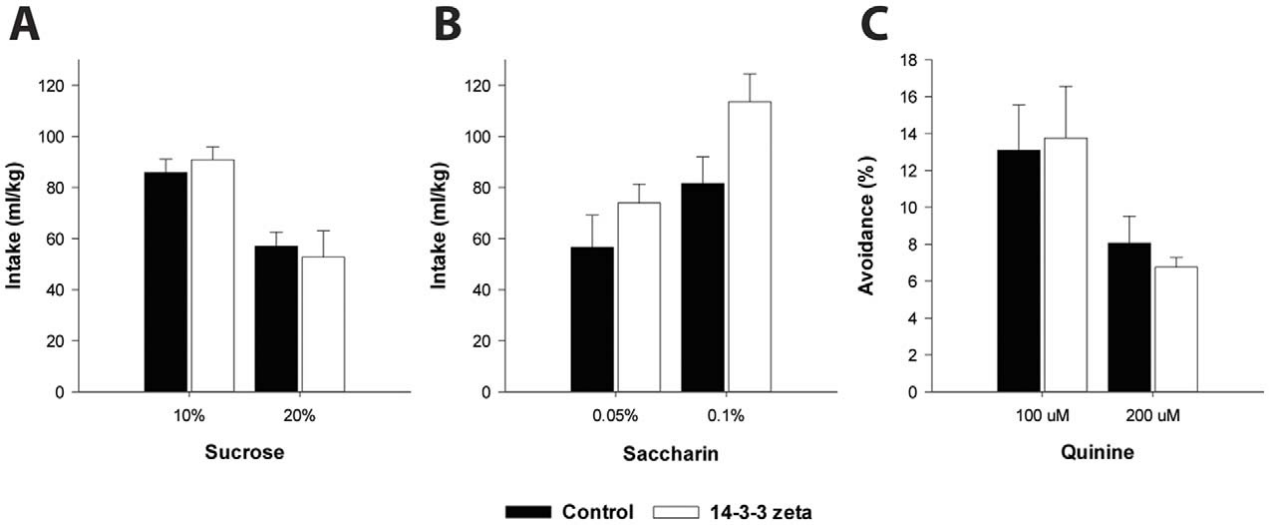
Taste control experiments show that the increase in alcohol intake in mice with CeA 14-3-3ζ knockdown is not secondary to altered taste sensitivity. Control mice and mice with CeA 14-3-3ζ knockdown showed equal intake of solutions containing *A* the caloric sweet tastant sucrose and *B* the non-caloric sweet tastant saccharin. *C* Aversion for the bitter tastant quinine was also not different between controls and mice with CeA 14-3-3ζ knockdown.

Finally, we also determined specificity of these findings for alcohol and potential confounding effects of 14-3-3ζ knockdown on taste sensitivity. Mice treated with the 14-3-3ζ 1854 shRNA showed equal intake of the caloric sweet tastant sucrose (Fig. 5*A*; F_group(1,11)_ = 0.002, N.S.; F_sucrose x group(1,11)_ = 1.04, N.S.) and the non-caloric sweet tastant saccharin (Fig. 5*B*; F_group(1,11)_ = 2.5, N.S.; F_saccharin x group(1,11)_ = 1.6, N.S.) as compared to control mice. The groups also showed similar preference for these sweet solutions (not shown). Mice with 14-3-3ζ knockdown also did not differ from control mice in aversion for bitter quinine solutions (Fig. 5*C*; F_group(1,11)_ = 0.001, N.S.; F_quinine x group(1,11)_ = 0.21, N.S.). Taken together, the increase in alcohol intake in mice with CeA 14-3-3ζ knockdown does not generalize to natural rewards and can not be explained by altered taste sensitivity.

Bilateral infection of the CeA was confirmed by post-mortem immunohistochemistry for GFP with the main infection site located between −0.9 and −1.5 mm from bregma (Figure 4E) [36]. Mice that showed unilateral infection were excluded from further analysis; one mouse was excluded after histology revealed hydrocephalus. There was occasional infection of the basolateral nucleus of the amygdala and in the caudate putamen along the injection tract.

## Discussion

The development of alcoholism is a progressive process that invariably starts with casual, social drinking, which escalates to heavy drinking, problem drinking and ultimately alcohol addiction. Consistent with its role in processing negative emotional stimuli, the amygdala contributes to alcohol consumption in human alcoholics and alcohol-dependent animals that display enhanced negative affect [19]–[22]. However, the amygdala is also important for perception of positive emotions [17] and it may therefore also be involved in the reinforcing properties of alcohol [57] and in escalation of alcohol intake in non-dependent animals [30]. Here, we combined gene expression analysis, RNA interference and a murine model for escalation of voluntary alcohol intake to identify amygdala 14-3-3ζ as a novel key modulator in the development of high alcohol intake.

### Amygdala 14-3-3ζ and escalation of alcohol intake

In the limited access choice paradigm, C57BL/6J mice show a rapid increase in alcohol intake in 7–10 days time. This rapid acquisition of alcohol consumption is likely driven by the positive subjective properties of alcohol. After two weeks of daily alcohol consumption, the mice reach their highest levels of alcohol intake and by that time, C57BL6/J mice also display inflexible alcohol intake in that they are insensitive to quinine adulteration of the alcohol solution when this is the sole source of alcohol [33]. In subsequent weeks, C57BL/6J mice maintain high levels of alcohol intake and develop indifferent alcohol drinking, indicated by persistent intake from aversive, quinine-adulterated alcohol despite the availability of non-adulterated alcohol [33]. Although the level of alcohol exposure achieved in our rodent model may not be sufficient to induce a genuine state of alcohol addiction, the inflexible and indifferent alcohol drinking patterns displayed by our mice show remarkable similarities to compulsive alcohol drinking in human alcoholics [5860]. The limited access choice paradigm therefore models important aspects of alcoholism-like behavior.

The current microarray analysis compared gene expression levels at three stages of the development of alcoholism-like behavior: initial escalation of alcohol intake (1 week), the stage where high alcohol intake is reached and signs of alcoholism-like behavior emerge (2 weeks) and the stage of stable high alcohol intake (4 weeks). Our analysis revealed that gene expression changes in the amygdala occur predominantly during the initial rapid escalation of alcohol intake (1 week). These data suggest that the amygdala contributes to the positive subjective properties of alcohol, which is consistent with the known involvement of the amygdala in the generation and perception of stimuli with positive emotional valence [16]–[18].

One of the genes that stood out from our analysis of alcohol-regulated amygdala genes is 14-3-3ζ. Up-regulation of this gene during initial escalation of alcohol intake was confirmed by qPCR analysis in an independent batch of mice. Moreover, using RNA interference we demonstrate functional involvement of amygdala 14-3-3ζ in the development of high alcohol intake and alcoholism-like behavior. Local knockdown of 14-3-3ζ in the amygdala lead to increased alcohol intake and a greater propensity to develop inflexible alcohol intake. Together with the observed up-regulation of 14-3-3ζ in the amygdala, these findings suggest that 14-3-3ζ may serve to restrict alcohol intake.

The observed increase in alcohol intake after 14-3-3ζ knockdown in the amygdala was behaviorally specific for alcohol, as we did not observe alterations in preference for natural rewards, i.e. sucrose and saccharin or in sensitivity to the aversive taste of quinine. The enhanced alcohol intake after amygdala 14-3-3ζ knockdown, together with the up-regulation of amygdala 14-3-3ζ expression during the initial increase in alcohol intake suggests that the rewarding properties of alcohol are increased in the absence of 14-3-3ζ. This implies that 14-3-3ζ may be a protective factor against the development of alcoholism; up-regulation of 14-3-3ζ would be required to demonstrate this role. The augmentation of alcohol intake after amygdala 14-3-3ζ knockdown may also reflect impaired behavioral control. In fact, the increase in inflexible alcohol intake that we found is indicative of loss of control over alcohol intake. However, it is also possible that mice with amygdala 14-3-3ζ depletions consume more alcohol and as a consequence develop inflexible alcohol intake more rapidly than control mice.

The present findings demonstrate an important role of amygdala 14-3-3ζ in the escalation of alcohol intake: 14-3-3ζ levels in the amygdala are enhanced and 14-3-3ζ knockdown causes profound increases in alcohol intake in mice. Alcohol has previously been shown to alter 14-3-3ζ levels, although in contrast to our current findings only after extended alcohol exposure. For example, prolonged alcohol use in rats and in alcohol dependent mice induced increased 14-3-3ζ gene and protein expression in nucleus accumbens and amygdala [54], [55]. Moreover, a recent study showed reduced 14-3-3ζ in the motor cortex of human alcoholics, i.e. after extended alcohol abuse [56]. The apparent contrast in the directional changes in expression of 14-3-3ζ may reflect brain region dependency of 14-3-3ζ regulation by alcohol: while 14-3-3ζ is consistently up-regulated by alcohol use in limbic brain regions, the same gene may be down-regulated in cortical regions. Although gene and protein expression changes may not necessarily cause behavioral changes, these studies suggest involvement of 14-3-3ζ in alcohol intake after prolonged alcohol use. In conclusion, 14-3-3ζ pathways in the amygdala constitute important mechanisms that are engaged during the descent of casual alcohol intake into alcoholism-like behavior.

### Biological function of 14-3-3ζ in relation to alcohol consumption

14-3-3 proteins are adapter proteins, that have multiple and diverse binding partners [46], [47]. Upon binding, they can regulate the activity or subcellular localization of other proteins and thereby influence multiple cellular processes including signal transduction or the cell cycle. 14-3-3 Proteins are most widely studied for their involvement in cancer [47], [61], [62], but they have also been associated with neurological diseases such as Parkinson's disease [63]–[65]. The binding partners of 14-3-3 proteins are diverse and include transcription factors and signaling molecules. The molecular mechanisms through which 14-3-3ζ influences alcohol consumption are currently unknown. However, several binding partners of 14-3-3 proteins have been implicated in alcoholism and may contribute to 14-3-3 modulation of alcohol intake.

For example, 14-3-3ζ is known to interact with protein kinase C isoforms (PKCs) including PKCε [48]. In fact, 14-3-3 was first identified as a PKC inhibitor [66], although later reports showed that 14-3-3 may enhance PKC activity [48], [67]. PKCs, including PKCδ and PKCε, are critically involved in alcohol sensitivity [52], [53], [68]. In fact, amygdala PKCε is important for alcohol intake [30]. Interactions of 14-3-3ζ with PKCs may contribute to these effects.

14-3-3 proteins may also affect GABAergic neurotransmission. Interactions of GABA-B receptor subunits with 14-3-3 proteins were reported [69]. Further, 14-3-3 can affect the phosphorylation of GABA-A receptor subunits [70] and GABA, in turn, can regulate 14-3-3 proteins [71]. Multiple studies have shown an association of alcohol dependence to genes encoding GABA-A receptor subunits alpha1 [45], [72]-[76] and alcohol is known to regulate GABA-A receptor subunits both *in vitro* and *in vivo* [77]–[81], possibly through 14-3-3ζ interactions with GABA-A receptors.

Interactions of 14-3-3 proteins with ionotropic glutamate receptors [49] are another candidate mechanism through which 14-3-3 proteins may affect alcoholism. AMPA receptors have been associated with alcoholism and alcohol intake in animal models [44], [50], [51]. 14-3-3ζ may modulate alcohol consumption by interacting with and affecting the activity of glutamate receptors.

Our microarray analysis revealed that, in addition to 14-3-3ζ, the GABA-A β3 subunit (Gabrb3) and the AMPA receptor α3 subunit (GRIA3) were also up-regulated in the amygdala during initial escalation of alcohol intake, suggesting that GABA-A and AMPA receptors in the amygdala are also involved in escalation of alcohol intake, possibly through interactions with 14-3-3ζ. Clearly, the elucidation of the molecular mechanisms and binding partners involved in 14-3-3ζ control over alcohol intake, both during initiation and maintenance of alcohol use, should be addressed in future research. In fact, the recent development of drugs that specifically interact with certain 14-3-3 complexes [82], [83] underscores the need to identify the 14-3-3ζ-protein complex that governs the escalation of alcohol intake.

### Conclusion

This study identifies amygdala 14-3-3ζ as a novel key modulator of alcohol intake. Interactions of 14-3-3ζ with signaling proteins such as PKCε [48] or neurotransmitter receptors [49] may contribute to these effects. The recent development of 14-3-3 complex specific drugs provides exciting opportunities to develop innovative treatment strategies for alcoholism.

## References

[1] WHO (2004). Global status report on alcohol.

[2] O'Brien CP (2008). Review. evidence-based treatments of addiction.. Philos Trans R Soc Lond B Biol Sci.

[3] Koob GF, Kenneth Lloyd G, Mason BJ (2009). Development of pharmacotherapies for drug addiction: A rosetta stone approach.. Nat Rev Drug Discov.

[4] van den Brink W (2012). Evidence-based pharmacological treatment of substance use disorders and pathological gambling.. http://dx.doi.org/10.2174/1874212221267084737.

[5] Koob GF, Ahmed SH, Boutrel B, Chen SA, Kenny PJ (2004). Neurobiological mechanisms in the transition from drug use to drug dependence.. Neurosci Biobehav Rev.

[6] Everitt BJ, Robbins TW (2005). Neural systems of reinforcement for drug addiction: From actions to habits to compulsion.. Nat Neurosci.

[7] Vanderschuren LJMJ, Everitt BJ (2005). Behavioral and neural mechanisms of compulsive drug seeking.. Eur J Pharmacol.

[8] Robinson TE, Berridge KC (2003). Addiction.. Annu Rev Psychol.

[9] Volkow ND, Li TK (2004). Drug addiction: The neurobiology of behaviour gone awry.. Nat Rev Neurosci.

[10] Bechara A (2005). Decision making, impulse control and loss of willpower to resist drugs: A neurocognitive perspective.. Nat Neurosci.

[11] Perry JL, Carroll ME (2008). The role of impulsive behavior in drug abuse.. Psychopharmacology (Berl).

[12] Sah P, Faber ES, Armentia LD, Power J (2003). The amygdaloid complex: Anatomy and physiology.. Physiol Rev.

[13] Phillips AG, Ahn S, Howland JG (2003). Amygdalar control of the mesocorticolimbic dopamine system: Parallel pathways to motivated behavior.. Neurosci Biobehav Rev.

[14] Geisler S, Zahm DS (2005). Afferents of the ventral tegmental area in the rat-anatomical substratum for integrative functions.. J Comp Neurol.

[15] Rodaros D, Caruana DA, Amir S, Stewart J (2007). Corticotropin-releasing factor projections from limbic forebrain and paraventricular nucleus of the hypothalamus to the region of the ventral tegmental area.. Neuroscience.

[16] Holland PC, Gallagher M (2004). Amygdala-frontal interactions and reward expectancy.. Curr Opin Neurobiol.

[17] Baxter MG, Murray EA (2002). The amygdala and reward.. Nat Rev Neurosci.

[18] Cardinal RN, Parkinson JA, Hall J, Everitt BJ (2002). Emotion and motivation: The role of the amygdala, ventral striatum, and prefrontal cortex.. Neurosci Biobehav Rev.

[19] Makris N, Oscar-Berman M, Jaffin SK, Hodge SM, Kennedy DN (2008). Decreased volume of the brain reward system in alcoholism.. Biol Psychiatry.

[20] Schneider F, Habel U, Wagner M, Franke P, Salloum JB (2001). Subcortical correlates of craving in recently abstinent alcoholic patients.. Am J Psychiatry.

[21] Schulteis G, Markou A, Cole M, Koob GF (1995). Decreased brain reward produced by ethanol withdrawal.. Proc Natl Acad Sci U S A.

[22] Koob GF (2003). Neuroadaptive mechanisms of addiction: Studies on the extended amygdala.. Eur Neuropsychopharmacol.

[23] Roberto M, Madamba SG, Stouffer DG, Parsons LH, Siggins GR (2004). Increased GABA release in the central amygdala of ethanol-dependent rats.. J Neurosci.

[24] Phelps EA, LeDoux JE (2005). Contributions of the amygdala to emotion processing: From animal models to human behavior.. Neuron.

[25] Everitt BJ, Parkinson JA, Olmstead MC, Arroyo M, Robledo P (1999). Associative processes in addiction and reward. the role of amygdala-ventral striatal subsystems.. Ann N Y Acad Sci.

[26] See RE (2005). Neural substrates of cocaine-cue associations that trigger relapse.. Eur J Pharmacol.

[27] Balleine BW, Killcross S (2006). Parallel incentive processing: An integrated view of amygdala function.. Trends Neurosci.

[28] Heilig M, Koob GF (2007). A key role for corticotropin-releasing factor in alcohol dependence.. Trends Neurosci.

[29] Roberto M, Cruz MT, Gilpin NW, Sabino V, Schweitzer P (2010). Corticotropin releasing factor-induced amygdala gamma-aminobutyric acid release plays a key role in alcohol dependence.. Biol Psychiatry.

[30] Lesscher HMB, Wallace MJ, Zeng L, Wang V, Deitchman JK (2009). Amygdala protein kinase C epsilon controls alcohol consumption.. Genes Brain Behav.

[31] Ahmed SH (2011). The science of making drug-addicted animals.. http://dx.doi.org/10.1016/j.neuroscience.2011.08.014.

[32] Lesscher HMB, Kas MJ, van der ES, van Lith HA, Vanderschuren LJMJ (2009). A grandparent-influenced locus for alcohol preference on mouse chromosome 2.. Pharmacogenet Genomics.

[33] Lesscher HMB, van Kerkhof LWM, Vanderschuren LJMJ (2010). Inflexible and indifferent alcohol drinking in male mice.. Alcohol Clin Exp Res.

[34] Ford MM, Yoneyama N, Strong MN, Fretwell A, Tanchuck M (2008). Inhibition of 5alpha-reduced steroid biosynthesis impedes acquisition of ethanol drinking in male C57BL/6J mice.. Alcohol Clin Exp Res.

[35] Rhodes JS, Best K, Belknap JK, Finn DA, Crabbe JC (2005). Evaluation of a simple model of ethanol drinking to intoxication in C57BL/6J mice.. Physiol Behav.

[36] Paxinos G, Franklin KB (2001). The mouse brain in stereotaxic coordinates..

[37] Roepman P, Wessels LF, Kettelarij N, Kemmeren P, Miles AJ (2005). An expression profile for diagnosis of lymph node metastases from primary head and neck squamous cell carcinomas.. Nat Genet.

[38] Phillips J, Eberwine JH (1996). Antisense RNA amplification: A linear amplification method for analyzing the mRNA population from single living cells.. Methods.

[39] Yang YH, Dudoit S, Luu P, Lin DM, Peng V (2002). Normalization for cDNA microarray data: A robust composite method addressing single and multiple slide systematic variation.. Nucleic Acids Res.

[40] Wu H, Kerr K, Cui X, Churchill GA, Parmigiani G, Garett ES, Izizarry RA, Zeger SL (2003). MAANOVA: A software package for the analysis of spotted cDNA microarray experiments.. The Analysis of Gene Expression Data: methods and software.

[41] Ernst J, Bar-Joseph Z (2006). STEM: A tool for the analysis of short time series gene expression data.. BMC Bioinformatics.

[42] Schmittgen TD, Livak KJ (2008). Analyzing real-time PCR data by the comparative C(T) method.. Nat Protoc.

[43] de Backer MW, la Fleur SE, Brans MA, van Rozen AJ, Luijendijk MC (2011). Melanocortin receptor-mediated effects on obesity are distributed over specific hypothalamic regions.. Int J Obes (Lond).

[44] Sanchis-Segura C, Borchardt T, Vengeliene V, Zghoul T, Bachteler D (2006). Involvement of the AMPA receptor GluR-C subunit in alcohol-seeking behavior and relapse.. J Neurosci.

[45] Radel M, Vallejo RL, Iwata N, Aragon R, Long JC (2005). Haplotype-based localization of an alcohol dependence gene to the 5q34 {gamma}-aminobutyric acid type A gene cluster.. Arch Gen Psychiatry.

[46] van Heusden GP (2009). 14-3-3 proteins: Insights from genome-wide studies in yeast.. Genomics.

[47] Morrison DK (2009). The 14-3-3 proteins: Integrators of diverse signaling cues that impact cell fate and cancer development.. Trends Cell Biol.

[48] Saurin AT, Durgan J, Cameron AJ, Faisal A, Marber MS (2008). The regulated assembly of a PKCepsilon complex controls the completion of cytokinesis.. Nat Cell Biol.

[49] Altar CA, Vawter MP, Ginsberg SD (2009). Target identification for CNS diseases by transcriptional profiling.. Neuropsychopharmacology.

[50] Breese CR, Freedman R, Leonard SS (1995). Glutamate receptor subtype expression in human postmortem brain tissue from schizophrenics and alcohol abusers.. Brain Res.

[51] Haugbol SR, Ebert B, Ulrichsen J (2005). Upregulation of glutamate receptor subtypes during alcohol withdrawal in rats.. Alcohol Alcohol.

[52] Hodge CW, Mehmert KK, Kelley SP, McMahon T, Haywood A (1999). Supersensitivity to allosteric GABA(A) receptor modulators and alcohol in mice lacking PKCepsilon.. Nat Neurosci.

[53] Olive MF, Mehmert KK, Messing RO, Hodge CW (2000). Reduced operant ethanol self-administration and in vivo mesolimbic dopamine responses to ethanol in PKCepsilon-deficient mice.. Eur J Neurosci.

[54] Contet C, Gardon O, Filliol D, Becker JA, Koob GF (2011). Identification of genes regulated in the mouse extended amygdala by excessive ethanol drinking associated with dependence.. Addict Biol.

[55] Bell RL, Kimpel MW, Rodd ZA, Strother WN, Bai F (2006). Protein expression changes in the nucleus accumbens and amygdala of inbred alcohol-preferring rats given either continuous or scheduled access to ethanol.. Alcohol.

[56] Mackay RK, Colson NJ, Dodd PR, Lewohl JM (2011). Differential expression of 14-3-3 isoforms in human alcoholic brain.. Alcohol Clin Exp Res.

[57] Hyytia P, Koob GF (1995). GABAA receptor antagonism in the extended amygdala decreases ethanol self-administration in rats.. Eur J Pharmacol.

[58] American Psychiatric Association (2000). Diagnostic and statistical manual of mental disorders (4th ed., text rev.)..

[59] Soo Hoo GW, Hinds RL, Dinovo E, Renner SW (2003). Fatal large-volume mouthwash ingestion in an adult: A review and the possible role of phenolic compound toxicity.. J Intensive Care Med.

[60] Leon DA, Saburova L, Tomkins S, Andreev E, Kiryanov N (2007). Hazardous alcohol drinking and premature mortality in russia: A population based case-control study.. Lancet.

[61] Macha MA, Matta A, Chauhan S, Siu KM, Ralhan R (2010). 14-3-3 zeta is a molecular target in guggulsterone induced apoptosis in head and neck cancer cells.. BMC Cancer.

[62] Bergamaschi A, Katzenellenbogen BS (2011). Tamoxifen downregulation of miR-451 increases 14-3-3zeta and promotes breast cancer cell survival and endocrine resistance.. http://dx.doi.org/10.1038/onc.2011.223.

[63] Ubl A, Berg D, Holzmann C, Kruger R, Berger K (2002). 14-3-3 protein is a component of lewy bodies in parkinson's disease-mutation analysis and association studies of 14-3-3 eta.. Brain Res Mol Brain Res.

[64] Yacoubian TA, Slone SR, Harrington AJ, Hamamichi S, Schieltz JM (2010). Differential neuroprotective effects of 14-3-3 proteins in models of parkinson's disease.. Cell Death Dis.

[65] Nichols RJ, Dzamko N, Morrice NA, Campbell DG, Deak M (2010). 14-3-3 binding to LRRK2 is disrupted by multiple parkinson's disease-associated mutations and regulates cytoplasmic localization.. Biochem J.

[66] Toker A, Ellis CA, Sellers LA, Aitken A (1990). Protein kinase C inhibitor proteins. purification from sheep brain and sequence similarity to lipocortins and 14-3-3 protein.. Eur J Biochem.

[67] Dai JG, Murakami K (2003). Constitutively and autonomously active protein kinase C associated with 14-3-3 zeta in the rodent brain.. J Neurochem.

[68] Choi DS, Wei W, Deitchman JK, Kharazia VN, Lesscher HM (2008). Protein kinase cdelta regulates ethanol intoxication and enhancement of GABA-stimulated tonic current.. J Neurosci.

[69] Couve A, Kittler JT, Uren JM, Calver AR, Pangalos MN (2001). Association of GABA(B) receptors and members of the 14-3-3 family of signaling proteins.. Mol Cell Neurosci.

[70] Qian Z, Micorescu M, Yakhnitsa V, Barmack NH (2012). Climbing fiber activity reduces 14-3-3-theta regulated GABA(A) receptor phosphorylation in cerebellar purkinje cells.. Neuroscience.

[71] Lancien M, Roberts MR (2006). Regulation of arabidopsis thaliana 14-3-3 gene expression by gamma-aminobutyric acid.. Plant Cell Environ.

[72] Dick DM, Plunkett J, Wetherill LF, Xuei X, Goate A (2006). Association between GABRA1 and drinking behaviors in the collaborative study on the genetics of alcoholism sample.. Alcohol Clin Exp Res.

[73] Sander T, Ball D, Murray R, Patel J, Samochowiec J (1999). Association analysis of sequence variants of GABA(A) alpha6, beta2, and gamma2 gene cluster and alcohol dependence.. Alcohol Clin Exp Res.

[74] Park CS, Park SY, Lee CS, Sohn JW, Hahn GH (2006). Association between alcoholism and the genetic polymorphisms of the GABAA receptor genes on chromosome 5q33-34 in korean population.. J Korean Med Sci.

[75] Covault J, Gelernter J, Hesselbrock V, Nellissery M, Kranzler HR (2004). Allelic and haplotypic association of GABRA2 with alcohol dependence.. Am J Med Genet B Neuropsychiatr Genet.

[76] Noble EP, Zhang X, Ritchie T, Lawford BR, Grosser SC (1998). D2 dopamine receptor and GABA(A) receptor beta3 subunit genes and alcoholism.. Psychiatry Res.

[77] Ravindran CR, Ticku MK (2006). Tyrosine kinase phosphorylation of GABA(A) receptor alpha1, beta2 and gamma2 subunits following chronic intermittent ethanol (CIE) exposure of cultured cortical neurons of mice.. Neurochem Res.

[78] Roberto M, Madamba SG, Moore SD, Tallent MK, Siggins GR (2003). Ethanol increases GABAergic transmission at both pre- and postsynaptic sites in rat central amygdala neurons.. Proc Natl Acad Sci U S A.

[79] Kerns RT, Ravindranathan A, Hassan S, Cage MP, York T (2005). Ethanol-responsive brain region expression networks: Implications for behavioral responses to acute ethanol in DBA/2J versus C57BL/6J mice.. J Neurosci.

[80] Rodd ZA, Kimpel MW, Edenberg HJ, Bell RL, Strother WN (2008). Differential gene expression in the nucleus accumbens with ethanol self-administration in inbred alcohol-preferring rats.. Pharmacol Biochem Behav.

[81] Anderson NJ, Daunais JB, Friedman DP, Grant KA, McCool BA (2007). Long-term ethanol self-administration by the nonhuman primate, macaca fascicularis, decreases the benzodiazepine sensitivity of amygdala GABA(A) receptors.. Alcohol Clin Exp Res.

[82] Wu H, Ge J, Yao SQ (2010). Microarray-assisted high-throughput identification of a cell-permeable small-molecule binder of 14-3-3 proteins.. Angew Chem Int Ed Engl.

[83] Rose R, Erdmann S, Bovens S, Wolf A, Rose M (2010). Identification and structure of small-molecule stabilizers of 14-3-3 protein-protein interactions.. Angew Chem Int Ed Engl.

